# Salivary proteomics profiling reveals potential biomarkers for chronic kidney disease: a pilot study

**DOI:** 10.3389/fmed.2024.1302637

**Published:** 2025-01-17

**Authors:** Bianca Uliana Picolo, Nathália Rabello Silva, Mário Machado Martins, Hebréia Oliveira Almeida-Souza, Letícia Cristina Machado de Sousa, Richard Costa Polveiro, Luiz Ricardo Goulart Filho, Robinson Sabino-Silva, Vivian Alonso-Goulart, Luciana Saraiva da Silva

**Affiliations:** ^1^Laboratory of Nanobiotechnology Prof. Dr. Luiz Ricardo Goulart Filho, Institute of Biotechnology, Federal University of Uberlândia, Uberlândia, Brazil; ^2^Faculty of Medicine, Federal University of Uberlândia, Uberlândia, Brazil; ^3^Faculty of Veterinary Medicine and Animal Science, Federal University of Uberlândia, Uberlândia, Brazil; ^4^Innovation Center in Salivary Diagnostic and Nanobiotechnology, Institute of Biomedical Sciences, Federal University of Uberlândia, Uberlândia, Brazil

**Keywords:** chronic kidney disease, LC–MS/MS, salivary proteomic, biomarker, PI-PLC, Sgsm2, API5

## Abstract

**Introduction:**

Chronic kidney disease (CKD) is a global public health problem, and the absence of reliable and accurate diagnostic and monitoring tools contributes to delayed treatment, impacting patients’ quality of life and increasing treatment costs in public health. Proteomics using saliva is a key strategy for identifying potential disease biomarkers.

**Methods:**

We analyzed the untargeted proteomic profiles of saliva samples from 20 individuals with end-stage kidney disease (ESKD) (*n* = 10) and healthy individuals (*n* = 10) using liquid chromatography–tandem mass spectrometry (LC–MS/MS) to identify potential biomarkers for CKD. A volcano plot was generated using a *p*-value of ≤0.05 and a fold change (FC) ≥ 2.0. Multivariate analysis was performed to generate the orthogonal partial least squares discriminant analysis (OPLS-DA) model and the variable importance in projection (VIP) scores. The accuracy of candidate biomarker proteins was evaluated using receiver operating characteristic (ROC) curves.

**Results:**

In total, 431 proteins were identified in the salivary proteomic profile, and 3 proteins were significantly different between the groups: apoptosis inhibitor 5 (API5), phosphoinositide phospholipase C (PI-PLC), and small G protein signaling modulator 2 (Sgsm2). These proteins showed good accuracy based on the ROC curve and a VIP score of >2.0. During pathway enrichment, PI-PLC participates in the synthesis of IP3 and IP4 in the cytosol. Gene ontology (GO) analysis revealed data on molecular functions, biological processes, cellular components, and protein classes.

**Conclusion:**

We can conclude that the salivary API5, PI-PLC, and Sgsm2 can be potential biomarker candidates for CKD detection. These proteins may participate in pathways related to renal fibrosis and other associated diseases, such as mineral and bone disorders.

## Introduction

Chronic kidney disease (CKD) is a complex disease that causes gradual renal dysfunction and can progress to end-stage kidney disease (ESKD) and associated morbidities such as cardiovascular disease (1). CKD is usually established by the presence of structural and/or functional kidney damage lasting at least 3 months through a low glomerular filtration rate (eGFR) and high levels of albumin (albuminuria) (2–4). ESKD is the most advanced CKD stage; it is irreversible and requires renal replacement therapy, such as dialysis or kidney transplantation (5).

An estimated 850 million people worldwide live with CKD (6). CKD is one of the most common diseases and a global public health problem with high prevalence and mortality rates (7, 8). In 2017, CKD caused 1.2 million deaths worldwide, and over 1.4 million deaths from cardiovascular diseases were attributable to impaired kidney function (9). CKD is silent in its early stages, with a frequent late diagnosis when the disease reaches later stages, increasing treatment costs and economic burden and predicting the worst outcomes (1, 6).

In clinical practice, serum creatinine and urinary albumin levels are used as biomarkers of renal function (10). However, these markers are inaccurate for the early detection of CKD and do not predict disease progression (10). A clinical challenge is identifying novel non-invasive biomarkers for CKD that allow early detection (11) and finding candidate biomarkers for CKD patients on hemodialysis may contribute to predicting and treating the progression of end-stage CKD, enabling early interventions and improving the quality of life of patients.

Omics proteomics is a tool that can be used for the biological investigation of chronic diseases and can provide stage-specific biomarkers (12), in addition to allowing the characterization of the expression, structure, functions, interactions, pathways, and modifications of proteins to better understand the molecular interactions underlying the pathogenesis of the disease (13, 14).

In the context of kidney disease, the field of identifying molecular biomarkers and protein profiles is growing, and the evidence is still inconclusive. Furthermore, published studies have considered different disease outcomes, such as renal cell carcinoma (15), renal calculi disease (16), diabetic kidney disease (17), and IgA nephropathy (18). The analyses were also performed using different equipment (MALDI-TOF-MS, LC–MS/MS, pressure cycling technology–pulse data-independent acquisition mass spectrometry, and CE-MS) and with different biological samples (blood and urine) or an animal model, which did not allow a comparison between them (15–18).

Another important aspect is the need for a non-invasive, reliable, and easy-to-collect method (19). Saliva is a body fluid with great potential, representing an increasingly valuable form of diagnosis and the search for biomarkers (19, 20).

Therefore, detecting candidate proteins as biomarkers for CKD in salivary samples has great potential in clinical nephrology practice, and proteomic analyses are an important investigative strategy. In this study, we used high-performance liquid chromatography–tandem mass spectrometry (LC–MS/MS) as an untargeted proteomic approach to identify salivary protein profiles and investigate their relationship with renal function in samples from patients with ESKD and healthy individuals. Pathway enrichment and gene ontology (GO) analyses of the salivary protein profiles of different groups were performed. The identification of differential protein profiles between the groups may have great potential for identifying novel pathways, contributing to advances in the detection of CKD, and improving the quality of life of these patients.

## Materials and methods

### Patients and study settings

This study received ethical approval from the Human Research Ethics Committee of the Federal University of Uberlândia (approval number 4.430.315.) All participants signed an informed consent form before participation. All experiments were performed in accordance with the relevant guidelines and regulations.

The inclusion criteria were as follows: > 18 years old; ESKD group with eGFR <15 mL/min/1.73m^2^, clinically diagnosed, and on hemodialysis for more than 1 year; healthy group with normal renal function (eGFR >60 mL/min/1.73m^2^), no comorbid medical conditions (hypertension and diabetes mellitus), no smoking, active physical activity, and good nutrition (higher consumption of fruits and vegetables and lower consumption of ultra-processed foods). The exclusion criteria were as follows: pregnant women and individuals with a history of alcohol and/or drug abuse or serious clinical conditions such as any type of cancer.

Twenty individuals participated in this pilot study; 10 had ESKD, and 10 were healthy. The ESKD patients were recruited at the Hemodialysis Sector of the Clinical Hospital of the Federal University of Uberlândia, and healthy individuals were recruited at the Ambulatory of the Clinical Hospital of the Federal University of Uberlândia, between December 2021 and July 2022.

### Sample collection

Sociodemographic (sex, age, and ethnicity/color) and biochemical (creatinine and eGFR) parameters were evaluated.

For saliva collection, patients rinsed their mouths to collect at least 1.5 mL of unstimulated total saliva using a saliva collector (Kolplast) according to the manufacturer’s instructions. The saliva was collected for a maximum of 5 min, and the sample was stored at −80°C until use.

Factors such as fasting time, diet, hydration level, medication use, and smoking can influence the characteristics of saliva, which also occurs with the serum. To reduce these variables, saliva collection was performed at rest (unstimulated saliva), with at least 1 h of fasting, without drinking or smoking, and with the patient in a comfortable state.

### Sample preparation and mass spectrometry analysis

In this study, we utilized the alpha-amylase depletion in saliva technique as described by Deutsch et al. (21). This is an effective method using potato extract, which promotes the selective adsorption of the enzyme onto potato starch, enriching other proteins in the sample. This approach significantly reduces alpha-amylase without affecting low-abundance proteins, making the salivary proteomic profile more accessible for biomarker detection.

Thus, alpha-amylase depletion was performed using soluble potato starch (Sigma S2004) (21, 22), with some modifications. A total of 100 mg of starch was reconstituted with 500 μL of distilled water and centrifuged at 13,000 × *g* for 5 min at room temperature (RT). The supernatant was removed, and 100 μL of saliva was added and centrifuged at 13,000 × *g* for 5 min at RT. Then, the supernatant was transferred to a new tube and stored at 4°C until use. The total protein concentrations of the samples were quantified using the PierceTM BCA Protein Assay Kit (Thermo Fisher Scientific Inc., Waltham, MA, USA), following the manufacturer’s instructions. For in-solution protein digestion, 50 μg of protein was treated with 1% de RapiGest SF (w/v) (Waters, Milford, MA), reduced with 0,5 M of dithiothreitol, alkylated with 0,5 M of iodoacetamide, and digested using trypsin (20 ng/μL) at 37°C, overnight. The peptide mixtures were desalted and concentrated using pipette tips with a C18 stationary phase (Omix, Agilent Technologies) according to the manufacturer’s instructions. Eluted peptides were processed using a vacuum concentrator and resuspended in 0.1% trifluoroacetic acid. The samples were analyzed using a liquid chromatograph (Agilent Infinity 1,260) and a high-resolution mass spectrometer equipped with an electrospray ionization source (Agilent 6,520 B Quadrupole Time-of-Flight - Q-TOF). For chromatography, an AdvanceBio Peptide Mapping column (Agilent Technologies) was used to separate the peptides in a 400-min multistep acetonitrile gradient at a flow rate of 0.4 mL/min. The ionization parameters were dry gas 8 L/min, dry temperature 325°C, nebulizer pressure 45 psi, and 4KV power applied in the capillary. Protein identification was carried out considering the mass of high resolution, mass error windows of less than 10 ppm, and the spectra mass/mass (MS/MS). To analyze the spectra, the Spectrum Mill MS Proteomics Workbench (Agilent Technologies) was used, with searches performed against the UniProt protein database, taxonomically restricted to *Homo sapiens* (human). The false discovery rate (FDR) was set to 1.2%. MassHunter Qualitative software v10.0 was used to process the raw data.

### Enrichment analysis

Enrichment analysis was performed using GO for the selected proteins, including biological process (BP), protein class (PC), molecular function (MF), and cellular component (CC), via Protein Analysis Through Evolutionary Relationships (PANTHER, http://pantherdb.org accessed on 23 February 2023) (23) The GO enrichment was performed using the most abundant proteins, present in more than 50% of the participants in each group, and the three main proteins (statistically significant) were candidate salivary biomarkers. Pathway enrichment analysis was performed using The Reactome Knowledgebase (https://reactome.org/, accessed on 23 February 2023) (24), and the *p*-value was corrected for FDR using the Benjamini–Hochberg method. After enrichment analyses and research in other scientific studies, we propose a hypothesis regarding the possible mechanisms of action of the identified proteins with significant differences between the groups.

### Statistical analysis

Characteristics of the ESKD patients and healthy controls were compared using the Student’s t-test and chi-square test. A Venn diagram was used to obtain an overview of the proteins identified in each group. Multivariate analysis was performed to generate an orthogonal partial least squares discriminant analysis (OPLS-DA) model. The variable importance in the projection (VIP) score was obtained based on the OPLS-DA model. The volcano plot was generated using a *p*-value of ≤0.05 and a fold change (FC) ≥ 2.0. The mean intensities of both groups of proteins identified as potential candidate biomarkers for CKD are presented in a heatmap. The accuracy of candidate biomarker proteins was evaluated using receiver operating characteristic (ROC) curves.

Statistical analyses were performed using Agilent Mass Profiler Professional (MPP) v.B.13.1.1, STATA 14.2, and MetaboAnalyst 5.0 (accessed on 23 February 2023). A 100% index was used for proteins present in at least one of the groups, and statistical analysis of the proteomics data was performed with values transformed into log10. Statistical significance was set at a *p*-value of <0.05.

## Results

### General characteristics of the study population

The baseline characteristics of the study population are shown in Table 1. The study included 10 healthy controls and 10 ESKD patients, 5 men and 5 women in each group. There were significant differences between the ESKD and healthy groups in terms of age, physical activity level, creatinine level, and eGFR.

**Table 1:** Baseline characteristics of the study participants, including the healthy and ESKD groups.

Parameters	Healthy group (*n* = 10)	ESKD group (*n* = 10)	*p*-value
Sociodemographic data			
Sex, female, *n* (%)	5 (50%)	5 (50%)	–
Age, year (mean)	40.0 ± 12.4	55.2 ± 8.5	**0.006**
Ethnicity/color, *n* (%)			0.147
White	4 (40%)	2 (20%)	
Black	1 (10%)	5 (50%)	
Brown	5 (50%)	3 (30%)	
Biochemical parameters			
Creatinine	0.96 ± 0.18	11.10 ± 3.29	**< 0.001**
eGFR	94.86 ± 27.07	4.70 ± 1.70	**< 0.001**

ESKD, end-stage kidney disease; eGFR, estimated glomerular filtration rate. Bold values indicate statistically significant differences between the healthy and ESKD groups.

### Salivary protein profile of the study population

**Figure 1:**
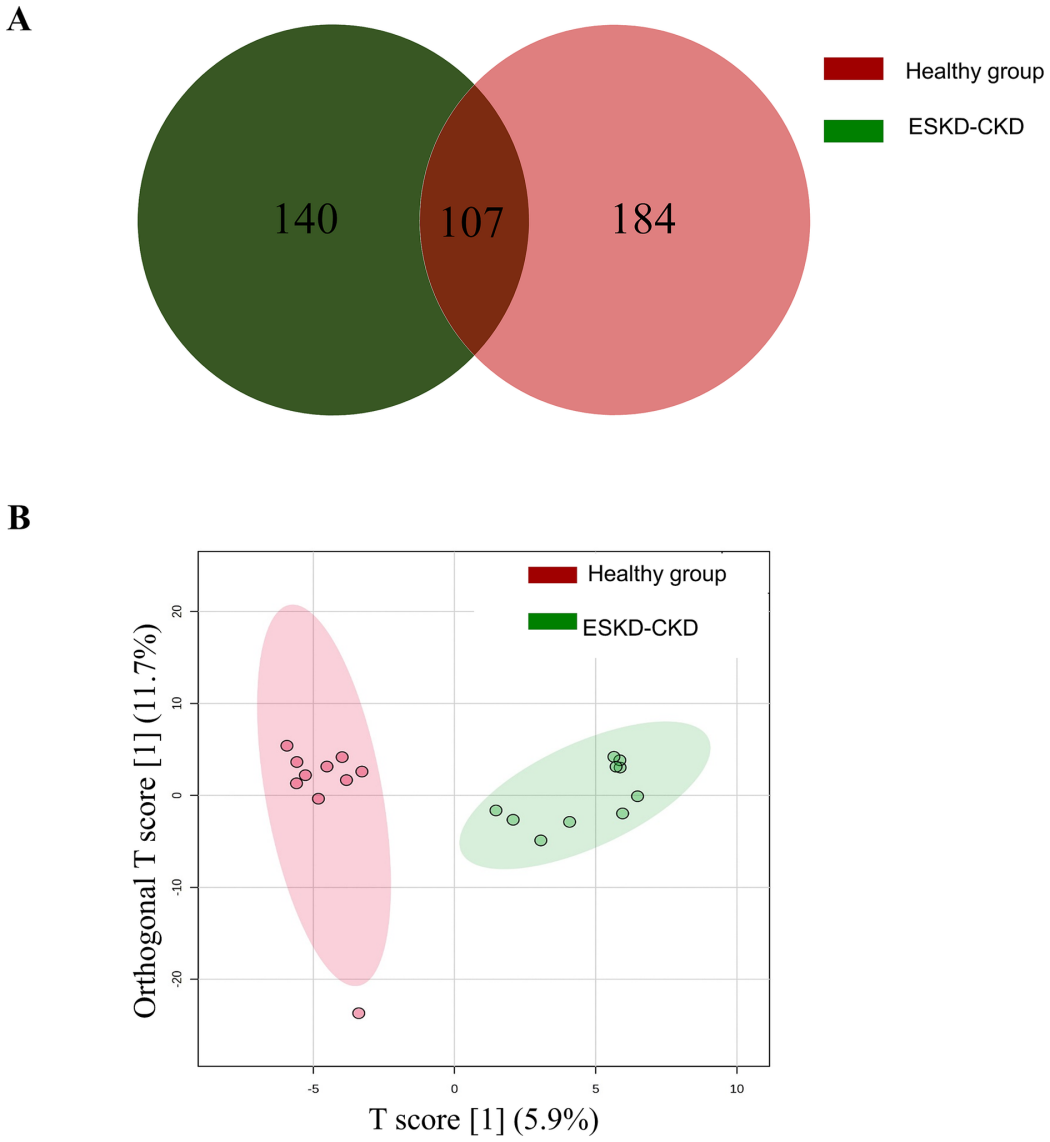
Characterization of the differentially identified proteins in human saliva samples of the healthy group and the ESKD group by mass spectrometry. **(A)** Venn diagram shows the number of proteins found in each group and the amount common between the groups. **(B)** The orthogonal projection to latent structure-discriminant analysis (OPLS-DA) score plots compared healthy group (red) and ESKD group (green).

In the present study, 431 proteins were identified. A Venn diagram revealed that 140 proteins were identified only in the ESKD group, 184 in the healthy group, and 107 in both groups (Figure 1A). Orthogonal projection to latent structure-discriminant analysis (OPLS-DA) revealed evident separation and clear clustering of the healthy group compared to the ESKD group (Figure 1B).

### Proteomic alterations in the ESKD group compared to the healthy group

**Figure 2:**
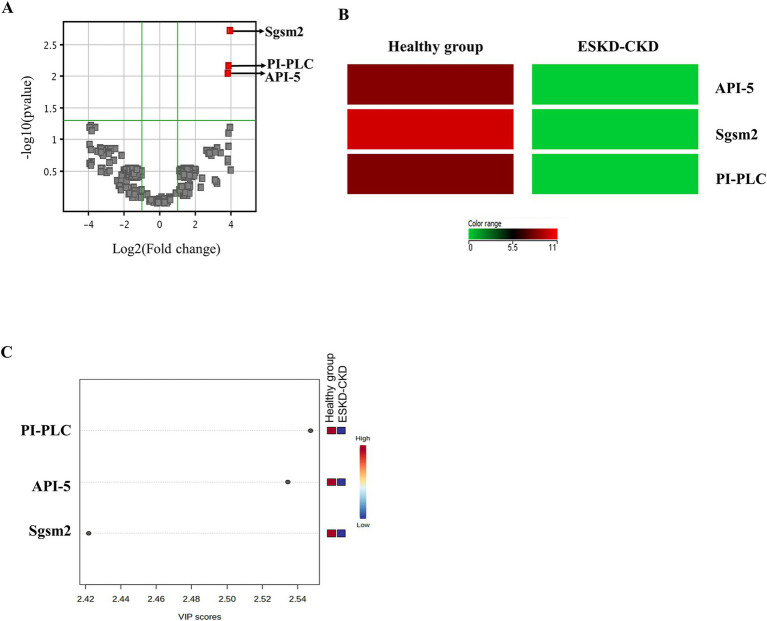
Proteins identified in the salivary proteomic profile with statistical differences between groups. **(A)** Volcano plot of all identified proteins. Red proteins show significant differences between groups (*p*-values ≤0.05 and fold change >2). Other proteins were colored in gray. **(B)** The heatmap shows the mean intensity of the three identified proteins with statistical differences between groups. The absence of intensity is represented by green, and the maximum intensity is represented by red. **(C)** Proteins with the highest variable importance in the projection (VIP) scores in the OPLS-DA model in the healthy and ESKD-CKD groups.

A comparison of the salivary proteomic profiles between the healthy and ESKD groups is shown in Figure 2. The volcano plot graph shows that the three proteins were significantly different between the healthy and ESKD groups (Figure 2A). These proteins are listed in Table 2.

**Table 2:** Characteristic of main proteins in the salivary proteome.

UniProt ID	Salivary proteome	Protein	Molecular weight (Da)	*p*-value	VIP score
O43147 SGSM2_HUMAN	Sgsm2	Small G protein signaling modulator 2	113,285	0.004	2.42174
Q9BZZ5 API5_HUMAN	API5	Apoptosis inhibitor 5	55	0.009	2.5344
Q86YW0 PLCZ1_HUMAN	PI-PLC	Phosphoinositide phospholipase C	70,411	0.009	2.5473

Based on the selection of the three most important proteins with differences between the groups, a heatmap analysis was performed to identify the difference in the intensity of these proteins between both groups (Figure 2B). Notably, these three proteins were present in the healthy group; however, these salivary proteins were not present in the ESKD group (Figure 2B). As expected, the three proteins detected in saliva also had the highest variable importance in the projection (VIP) scores (VIP >2), as determined by the OPLS-DA model (Figure 2C).

### GO enrichment of salivary proteomic and candidates to salivary biomarker and pathway enrichment analysis of proteins

**Figure 3:**
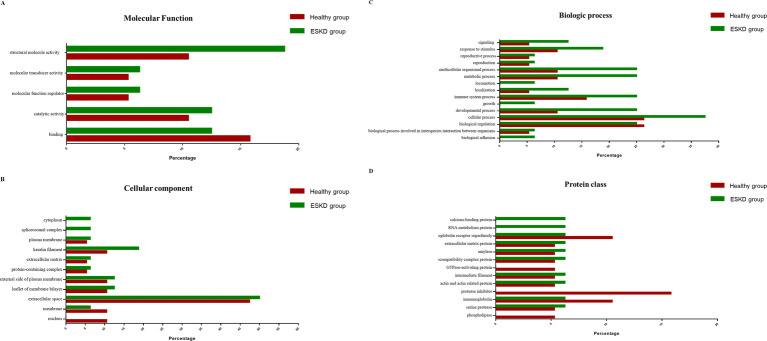
GO enrichment analysis of the proteins most abundant in at least 50% of individuals in the ESKD group (in red) and healthy group (green). **(A)** molecular function; **(B)** cellular component; **(C)** biological process; and **(D)** protein class.

Figure 3 shows the GO enrichment analysis of the most abundant proteins in the healthy and ESKD groups, present in at least 50% of the participants in each group. Regarding the MF, it is possible to observe that structural molecule activity, molecular transducer activity, molecular function regulator, and catalytic activity were higher in the ESKD group, while the function of binding was higher in the healthy group (Figure 3A). In the CC, the majority of salivary proteins in the ESKD group were present in the cytoplasm, spliceosomal complex, and keratin filaments. The presence of proteins in the membrane and nucleus was not identified in the ESKD group compared to the healthy group (Figure 3B). Notably, the BP was higher in the ESKD group, such as signaling, response to stimulus, multicellular organism process, metabolic process, locomotion, localization, immune system process, growth, developmental process, cellular process, and biological adhesion (Figure 3C), only the biological regulation was higher in the healthy group. Regarding PC, calcium-binding proteins and RNA metabolism proteins were higher in the ESKD group, and immunoglobulin receptor superfamily, protease inhibitor, immunoglobulin, and phospholipase levels were higher in the healthy group (Figure 3D). Other PCs, such as extracellular matrix proteins, amylases, major histocompatibility complex proteins, intermediate filaments, actin proteins, and serine proteases, were present in both groups.

**Figure 4:**
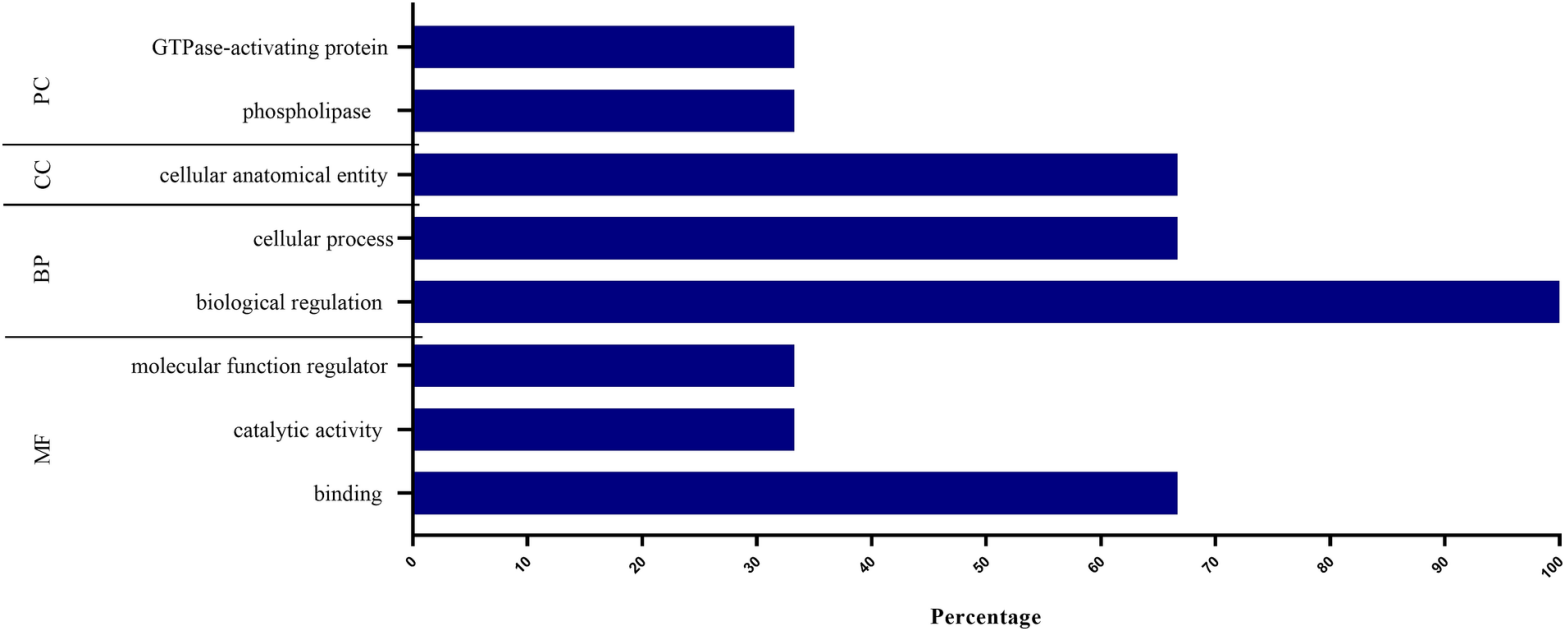
GO enrichment analysis of the three selected main proteins, with statistical differences between groups, in salivary protein profiling. PC, protein class; CC, cellular component; BP, biological process; MF, molecular function.

Figure 4 shows the GO enrichment analysis of the three protein candidates used as salivary biomarkers. The PC of the GO analysis revealed the phospholipase and GTPase-activating protein classes. Regarding the CC, a cellular anatomical entity was observed, indicating that proteins can be found in the nucleus or membrane. Biological regulations and cellular processes have been highlighted in the context of BP. In this case, we can say that the analyzed BP has functions such as the activation of GTPase activity, negative regulation of the apoptotic process, apoptotic process, cell activation, single fertilization, and positive regulation of cytosolic calcium ion concentration. For MF, binding functions, catalytic activity, and molecular function regulators were observed. Protein and RNA binding were involved, GTPase activity was involved in catalytic activity, and GTPase activator activity was involved in molecular function regulation.

When we analyzed the three protein candidates for salivary biomarkers that presented a statistical difference between the groups, it was shown that only PI-PLC had pathways identified by The Reactome Knowledgebase. In this case, PI-PLC participates in the synthesis of IP3 and IP4 in the cytosol.

### ROC curve analysis

**Figure 5:**
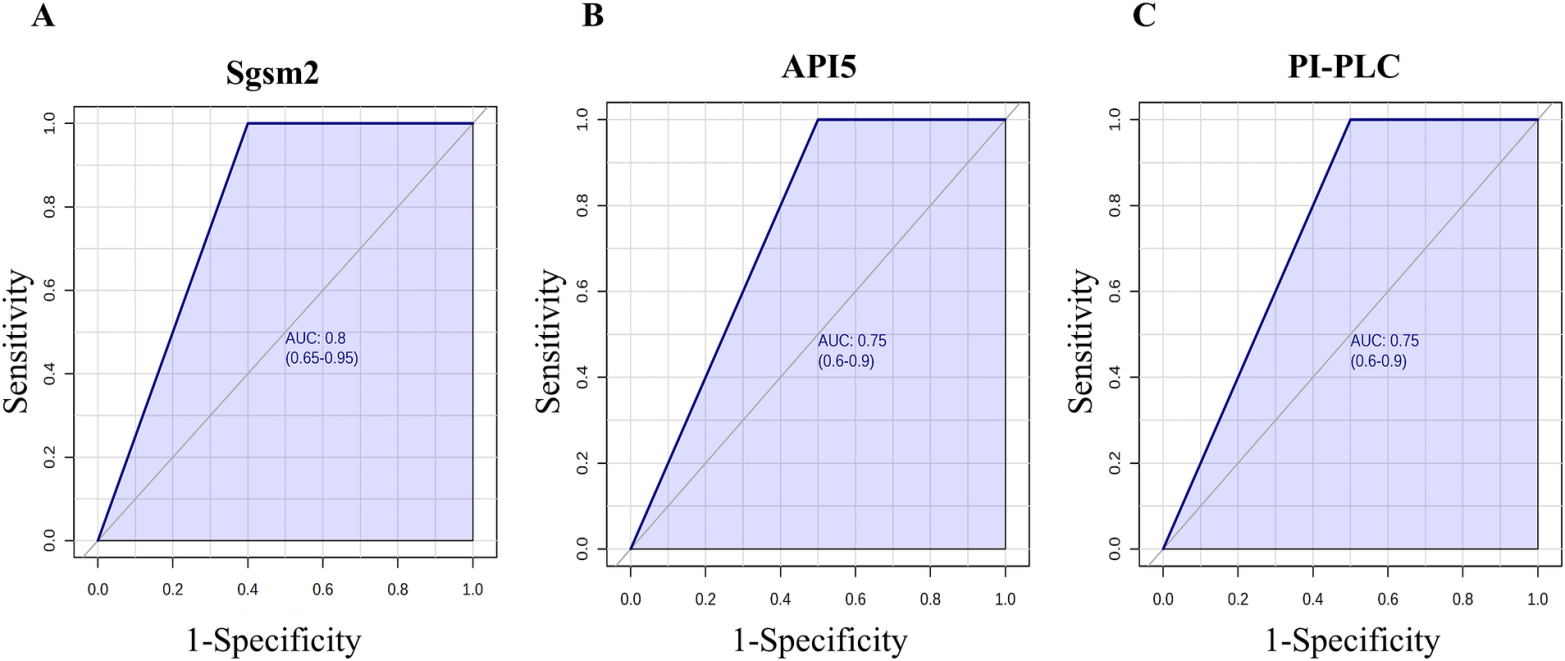
ROC curve of the three main proteins in salivary proteome **(A)** Sgsm2, **(B)** API5, and **(C)** PI-PLC.

The ROC curve analysis was performed to analyze the diagnostic potential of the three proteins with statistical differences between the groups (Figure 5). The AUC to discriminate ESKD patients and healthy subjects was 0.8 (CI 95% 0.65–0.95) for the salivary protein Sgsm2 (Figure 5A). The AUC was 0.75 (CI 95% 0.6–0.9) for salivary API5 and PI-PLC (Figures 5B,C, respectively). Thus, these three proteins presented adequate sensitivity and specificity to be considered potential salivary biomarkers for CKD.

## Discussion

In this study, we analyzed the proteomic profiles of saliva in patients with ESKD and healthy individuals using LC–MS/MS, which uses universal measurements for analysis (mass and charge) with high diagnostic potential. We found three proteins with statistically significant differences between the groups, which appeared only in the healthy group. The protein candidates as biomarkers for CKD were API5, PI-PLC, and Sgsm2, which presented high screening and detection potential for the disease. Pathway enrichment and gene ontology (GO) analyses were performed for three main proteins.

Studies have identified candidates for CKD biomarkers obtained through proteomics with the aim of early diagnosis and prognosis of CKD progression (14), especially with serum and urine samples (15, 18, 25–27). Proteins are the major constituents of saliva, and the salivary proteome at the time of sample collection represents the cellular function. The human salivary proteome has been well-characterized and is considered significant for diagnosis (28). A total of 2,290 proteins were compiled in saliva and compared to the plasma; the presence of these proteins in saliva is related to neurological, immune system, and ophthalmological diseases, which may indicate that saliva is a potential source of molecular markers for the design of non-invasive diagnostic strategies (19, 29).

Some studies have shown the use of the salivary proteome in different diseases, such as gastric cancer (30), type II diabetes (31), oral cancer (32), lupus erythematosus (33), and Sjögren’s syndrome (34). Regarding CKD, it has been shown that immunological and inflammatory salivary components, such as IgA, IgG, nitric oxide (NO), and C-reactive protein (CRP), are altered in patients with kidney disease undergoing hemodialysis compared to the healthy group. These alterations can be used to monitor the disease (35). Thus, using saliva samples for diagnosis has the advantage of being a non-invasive method with the potential to identify biomarkers for CKD.

Furthermore, salivary urea and creatinine levels in animal models and patients were higher in patients with advanced kidney disease and may be considered biomarkers of renal function (36). Several studies have shown the possibility of using salivary urea and/or creatinine as biomarkers for CKD (37–39). Techniques such as ATR-FITR can be used to identify potential biomarkers of CKD from salivary samples (40, 41).

However, to our knowledge, few studies have been conducted on salivary protein profiling and CKD. Tong et al. (42) identified candidate salivary biomarkers for CKD using magnetic beads and MALDI-TOF-MS in hemodialysis patients. Our study found three different proteins with statistical significance in the salivary proteome between the healthy and ESKD groups. The proteins found in the salivary proteome, PI-PLC, Sgsm2, and API5, were present in the healthy group but not in the ESKD group, and they can be considered candidates for CKD biomarkers with high diagnostic potential.

The biological processes analysis of salivary proteins in this study identified highly expressed processes in patients with CKD when compared to healthy individuals, such as signaling processes, responses to stimuli, metabolic processes, immune system processes, cell processes, development processes, and biological adhesion. These findings collaborate with what is found in the literature, for example, responses to immunological stimuli and immune and cellular system processes, such as adaptive and innate immune responses, which lead to increased inflammation in CKD (43, 44), response to oxidative stress (45, 46) and increased leukocyte migration and adhesion (47). In addition, there are studies that show that, in addition to these processes, there are proteins that participate in cell signaling pathways that lead to disease progression (46, 47). In our study, we also saw that the CKD group has higher calcium-binding protein. Schmidt et al. showed that Sparc-related modulate calcium-binding protein 2, as a potential biomarker for CKD, to estimate the prognosis and increased risk of disease progression in patients with CKD (48).

Furthermore, GO enrichment was analyzed only for proteins that are candidates for CKD biomarkers, and it was possible to identify that all three proteins participate in the biological regulation process. Two proteins participate in cellular processes and may be involved in cellular activation and the homeostasis process. These data agree with the pathophysiology of CKD, as the loss of important proteins for biological regulatory processes and mineral and bone homeostasis can be justified by the worsening of the disease. Thus, these data may be useful to identify pathways and processes activated in ESKD, allowing the design of prospective studies that explore the response to therapy.

**Figure 6:**
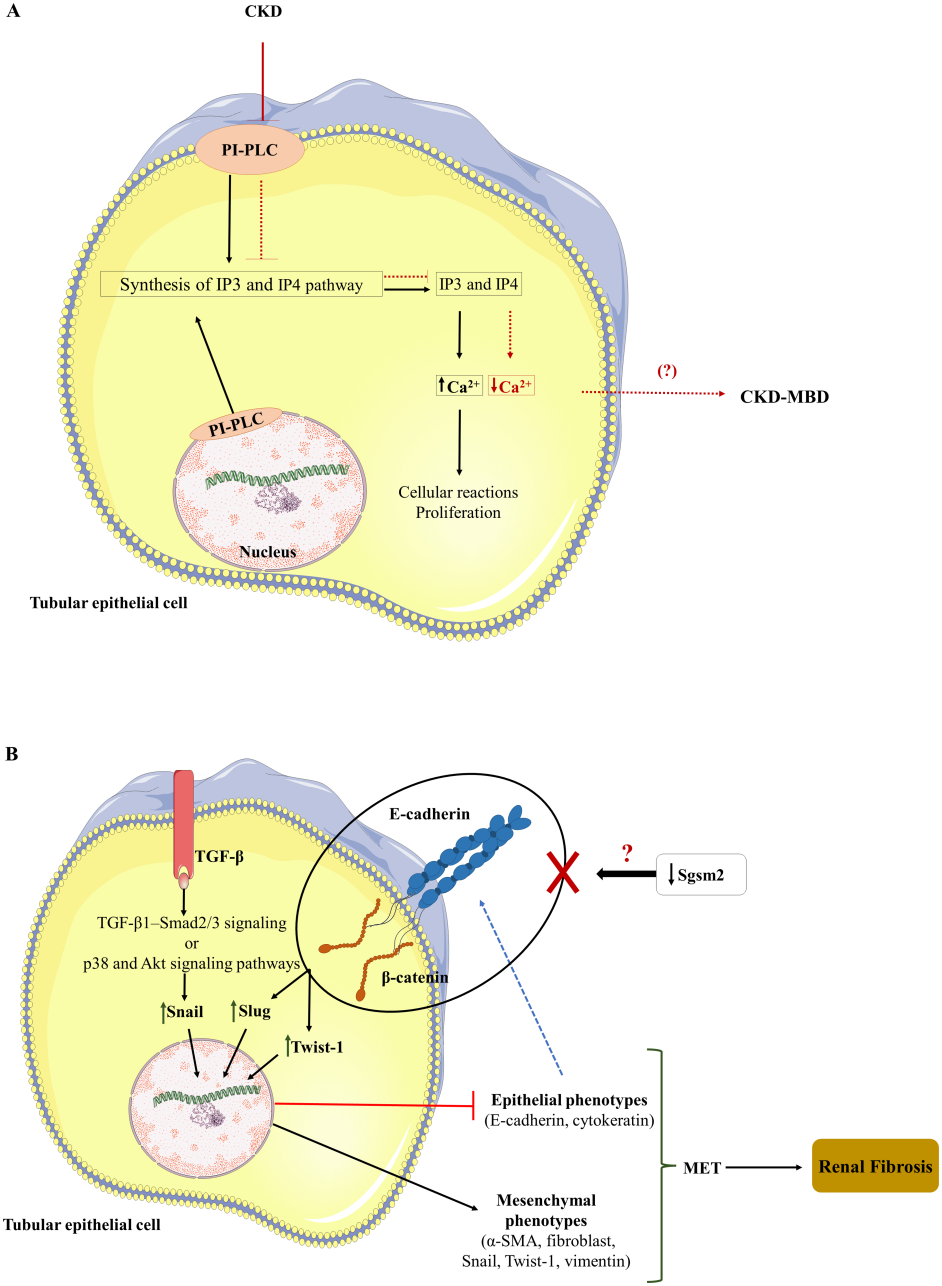
**(A)** The figure shows the PI-PLC protein, which participates in the IP3 and IP4 synthesis pathways and mobilizes calcium to regulate cell reactions and proliferation requiring free calcium. This study showed that the presence of CKD inhibited PI-PLC expression. As a result, the IP3 and IP4 synthesis signaling pathway is impaired, decreasing or inhibiting the expression of IP3 and IP4 decreasing the mobilization of intracellular calcium. It is hypothesized that this dysregulation of calcium mobilization may progress to CKD-MBD (arrows in red show pathway advancement from the CKD patient hypothesis). **(B)** The figure shows the increased expression of transcription factors such as Snail from the activation of TFG-β receptor-dependent pathways (TGF-β1-Smad2/3 and p38 and Akt), which lead to decreased epithelial phenotypes and increased mesenchymal phenotypes. When there is a decrease or no expression of epithelial markers, there is an increase in the expression of transcription factors, such as Slug and Twist-1, which also lead to the inhibition of the epithelial phenotype and increase of the mesenchymal phenotype. This change in the phenotypic profile promotes the epithelial–mesenchymal transition, which leads to renal fibrosis. This study showed that patients with CKD did not express the Sgsm2 protein, and the hypothesis is that the absence of this protein leads to a decrease in the expression of E-cadherin and b-catenin, increasing the expression of mesenchymal markers and decreasing epithelial markers favoring EMT and, consequently, increasing renal fibrosis.

Literature searches have shown that no studies are showing the relationship between API5 protein and kidney diseases. Protein PI-PLC plays an important role in signal transduction processes and is related to the regulation of intracellular Ca^2+^ (49). Furthermore, this enzyme belongs to the phospholipase C superfamily, as observed in the GO analyses (Figures 3, 4). The GO analyses revealed the presence of molecular functions that represent PI-PLC proteins, including biological regulation and cellular processes. This protein is present in the nucleus and cell membrane. Different subtypes of PI-PLC isozymes have highly conserved domains and can play different patterns and roles in organisms. These isozymes can be activated by G protein-coupled receptors or tyrosine kinases, leading to the activation of calcium regulation (50). The pathway enrichment analysis revealed that PI-PLC participated in the synthesis of IP3 and IP4 in the cytosol. The main function of IP3 and IP4 is to mobilize Ca^2+^ to regulate cellular reactions and proliferation, which require free calcium (51, 52). Calcium signaling is important in kidney epithelial cells. CKD is characterized by the disruption of bone and mineral metabolism, resulting in a complex called chronic kidney disease–mineral and bone disorder (CKD-MBD), which begins in the early stages of CKD and increases with disease progression (53). One of the characteristics of this disorder is the disturbance of calcium metabolism (54), and the lack of PI-PLC expression in patients with CKD-MBD can be associated with CKD-MBD (Figure 6A). The PI-PLC protein can be considered a candidate biomarker for ESKD, and in addition to being able to act in the development of new diagnostic tests, understanding the action of this enzyme in the disease signaling pathway can benefit the design of new specific therapies for kidney-related diseases.

The RUN and TBC1 domain-containing protein 1, also called small G protein signaling modulator 2 (Sgsm2), has GTPase activator activity. It regulates the G protein toward interaction via RAP and RAB (55), collaborating with our GO analysis findings presented in Figures 4D, 5, where it is possible to see the presence of the GTPase-activating protein class and binding, catalytic activity, and molecular function regulator in molecular function.

A study relating Sgsm2 with breast cancer showed that silencing the expression of Sgsm2 protein resulted in a decreased expression of epithelial markers such as E-cadherin, *β*-catenin, and Paxillin, in addition to increased expression of markers such as Snail and Twist-1. In that same study, it was possible to see a strong interaction of the Sgsm2 protein with E-cadherin/β-catenin cell junction complexes. A possible hypothesis generated by this study is that the sgsm2 protein coordinates cell adhesion and migration through an E-cadherin-mediated epithelial–mesenchymal transition (EMT) process during the initial stage of cancer migration (56). Cheng et al. showed that E-cadherin expression was decreased in ESKD patients with detrusor underactivity but not in ESKD patients with bladder oversensitivity (57).

Kidney fibrosis results from the progression of CKD. EMT, first described for tumor processes, has gained prominence in the renal area because it is a transitional process that leads epithelial cells to a fibroblast phenotype, leading to the evolution of fibrotic lesions in injured kidneys (58, 59). EMT is characterized by decreased expression of epithelial proteins such as E-cadherin, zone occludens (ZO-1), and cytokeratin and increased expression of mesenchymal markers such as vimentin, fibroblast, Snail, and Twist-1 (59). It was shown that capsaicin treatment was able to improve renal fibrosis by preventing the change in the phenotype of tubular epithelial cells through the inhibition of the TGF-β1–Smad2/3 signaling pathway *in vivo* and *in vitro*, increasing the expression of E-cadherin (60). The TGF-β1–Smad2/3 signaling can lead to renal fibrosis by the activation of Snail, and the *δ*-opioid receptor can act like an antifibrotic factor regulating the Snail gene from TGF-b/Smad, p38, and Akt signaling pathways (61).

Although the relationship between Sgsm2 and kidney disease has not been described in the literature, this protein may be related to the EMT that occurs in renal fibrosis in patients with CKD (Figure 6B). Thus, this protein can potentially serve as a salivary biomarker of ESKD. Understanding and designing a signaling pathway involving the Sgsm2 protein and CKD can be considered a way to build a diagnostic model, including this protein as a salivary biomarker for the disease. In this way, it would be possible to carry out the diagnosis of ESKD using a less invasive method and to adapt effective therapeutic proposals to try to regress the progression of the disease. Molecular validation of the findings of this study is necessary to develop theranostic platforms for Sgsm2 and kidney diseases.

Therefore, we consider that the method used, digestion in solution, and analysis in the HPLC system coupled with a high-resolution quadrupole time-of-flight (Q-TOF) mass spectrometer with electrospray ionization (ESI), allows for the separation of proteins and peptides covering a wide concentration range of different biological samples. In addition, the analysis of the most severe CKD group revealed protein profile changes compared to healthy individuals, which could lead to the identification of potential biomarkers for diagnosing the disease.

It is important to search for new biomarkers, with the aim of complementing and/or improving the diagnosis and therapy of CKD. In this study, we found proteins whose lack of expression are potential candidates for being biomarkers for CKD. Although the detection of diseases with biomarkers, whose absence of expression indicates disease progression, appears to be a limitation, in the case of CKD, the absence of a protein in a sample of patients may be directly associated with a decrease in the glomerular filtration rate, indicating that there is indeed progression of the disease to more advanced stages.

Recent studies, using the omics approach, show that patients with CKD may present a decrease in the expression of proteins or metabolites, in different biological samples, as CKD progresses to advanced stages (27, 62–64), and can still be considered potential biomarkers for the disease.

In the context of therapy, mapping the proteomic profile can provide more information about the pathophysiological processes associated with CKD, as well as the signaling pathways involved. This approach contributes to a deeper understanding of the disease, potentially leading to the identification of new therapeutic targets to improve renal function or personalize treatment based on the proteomic profile observed in the patient (65).

The diagnostic confirmation of CKD by new biomarkers can occur through tests that allow the identification of the presence or absence of protein, such as biosensors and lateral flow, which can be used in conjunction with markers currently used in clinical practice, to indicate the state of kidney function.

This study has some limitations. Although participants were selected carefully, the groups had different characteristics, which could lead to failures in data interpretation due to individual differences. Nevertheless, the individual profile reflects the reality in Brazil, and the prevalence of CKD is usually observed in older adults (66). Another limitation is the absence of replicates and the small sample size. Therefore, proteins as potential biomarkers need to be investigated in a larger cohort in longitudinal studies to better understand the mechanisms involved in CKD at the molecular level and to answer some questions about whether these findings are associated with long-term outcomes.

Finally, proteomic salivary profiling was used to identify differences between healthy individuals and patients with ESKD. Three candidate proteins for ESKD biomarkers were identified, with the two main biomarkers, PI-PLC and Sgsm2, having different functions; one is a phospholipase that regulates calcium reactions, and the other is a GTPase-activating protein. Sgsm2 has great potential to act as a regulator of the epithelial–mesenchymal transition process in renal fibrosis by decreasing the expression of epithelial markers and increasing the expression of mesenchymal proteins. PI-PLC is important in mobilizing Ca^2+^, which is responsible for cell function and proliferation. More studies are needed to understand the intrinsic mechanisms involved in CKD at the molecular level, and the findings of this study can contribute to future applications in the diagnostic and prognostic fields. Furthermore, future studies will be needed to investigate the correlation between these proteins and traditional markers, in addition to exploring their added diagnostic value, as substitutes or as complements to established biomarkers.

## Data Availability

The data presented in the study are deposited in the Proteomics Identification Database (PRIDE) repository, https://www.ebi.ac.uk/pride, accession number PXD045874.
