# Supplementary material for: Salivary proteomics profiling reveals potential biomarkers for chronic kidney disease: a pilot study
Source: Front Med (Lausanne). 2025 Jan 17;11:1302637. doi: 10.3389/fmed.2024.1302637 (PMC11784343; doi:10.3389/fmed.2024.1302637)
Supplement: Supplementary file 1 [file Data_Sheet_1.pdf]

Supplementary Table 1. Characterization of the 431 proteins found in the salivary proteomic profile of patients with CKD-ERSD and healthy subjects.

Columns Protein Observed	Spectra Protein Observed	Database Accession	%AA Coverage	Distinct Peptides
19	104	B7ZMD7	49.9	29
13	53	A1A4E9	50.8	24
7	11	P08779	20.5	8
6	9	P02533	12.2	5
4	4	A0A1B0GVI3	6	3
17	84	Q96K68	36.4	19
16	78	Q6N092	32.5	17
16	68	Q6MZV6	33.6	15
13	46	P0DOX2	27.4	13
12	19	Q6MZV7	13.5	6
11	17	A0A0B4J2B5	34.6	3
9	13	A0A5C2G624	27.4	3
8	11	A0A5C2GM19	27.4	3
9	13	A0A5C2GQU8	28.5	3
8	11	A0A890VXU4	27.2	3
8	12	A0A5C2G894	25.4	3
9	12	A0A5C2GHY4	25.1	3
13	40	B2RBS8	35.4	20
14	37	P01833	23.5	18
12	28	P48668	25	14
12	27	A0A0S2Z428	23.2	13
9	16	B4E1T1	14.9	9
6	10	P12035	6.2	4
3	3	H6VRG2	3.2	2
13	35	B4DRW1	26.1	13
11	17	A7Y9J9	2.7	12
10	35	P01036	70.9	9
8	29	P09228	38.2	6
9	26	P01037	51	6
12	28	Q0KKI6	39.2	9
2	2	A0A075B6P5	16.6	2
11	16	P12273	38.3	8
10	19	A0A140VK00	30.8	7
13	21	A0A804GS07	19.9	6
11	18	P01591	41.5	6
8	11	P04083	20.5	6
9	12	A0A5C2GJP2	18.8	2
11	16	A0A590UJ76	1.6	5
5	7	A0A0C4DGN4	22.6	4
11	28	A0A5E4	20.4	4
6	15	A0A0B4J231	22.3	4
9	10	Q9UGP3	30.3	2

9	11	A0A5C2GHF2	18.1	2
6	6	B4DUH8	14.7	3
12	18	A0A7S5BZE3	20.5	2
4	4	Q01546	4.2	3
1	3	P01871	7.9	3
9	10	A0A5C2GVB5	18.2	2
8	11	A0A5C2GET1	19.8	2
9	10	A0A5C2GE91	25.8	2
9	10	A0A7S5C2L3	19.3	2
8	10	A0A5C2GFH4	18.8	2
1	5	P15515	36.8	2
1	1	A0A024RBL6	1.3	1
9	10	A0A2U8J919	25.4	2
5	5	P27815-7	1.6	2
4	4	P04080	24.4	2
2	4	Q01955	2.5	2
4	7	Q68D51	2.2	2
1	2	A0A024R546	3.6	2
2	2	A0A248RGE3	14.4	2
5	5	A0A024R8D7	10.7	2
2	2	Q6P461	8.3	2
4	6	Q02388	0.8	2
2	2	A0A0B4J259	17.5	2
3	4	H6VRF8	3.2	2
1	1	B3KXN4	1.9	1
3	3	A0A5C2G1E5	23	2
3	3	A0A5C2GDF0	24	2
2	3	A0A5C2FU39	24	2
3	3	A0A5C2FT69	24	2
1	1	B0QYB2	9.5	1
5	12	A0A087X011	0.6	1
2	2	B7Z2L8	9.7	1
1	1	A0A140VK44	6.9	1
1	1	A0A2H4FZ19	10.4	1
6	13	P02814	26.5	1
3	3	F2Z2U4	0.4	2
1	1	A0A0A0MS03	7.8	1
2	2	A0A1L2BPJ1	5.9	1
9	15	A0A075B6Z2	38	1
2	2	F8WCR9	13.2	1
1	4	Q5T5X7	1	1
1	1	A0A087X1N3	2.1	1
2	2	A0A291FGE6	0.9	1
1	1	Q58EX2	0.4	1
2	3	A0A1W2PR06	3.5	1
2	2	Q8IVL5	0.9	1

6	10	A8KAL3	1.7	1
1	2	O60303	1.6	1
1	1	J3QLF6	40.4	1
1	1	A0A3Q8B178	0	1
1	1	Q15286-2	9.8	1
2	5	Q504W3	13	1
1	1	A0A5C2GC48	20.5	1
3	3	Q5CZC0	0.1	1
6	7	A0A1B0GUK8	6.3	1
1	1	G3V560	18.2	1
1	1	A0A087WUQ9	8.6	1
1	1	B4E209	1.6	1
2	5	Q9Y2J4	1.2	1
1	2	A0A2R3Z0D6	14.7	1
1	1	A0A024R4F1	2.7	1
1	1	A6PVD9	4.4	1
1	3	Q9NYA1-2	4	1
3	3	A0A024R9Y5	2.3	1
10	29	Q658W4	1.2	1
1	1	A0A024RE02	2.3	1
1	1	A5YKK6	0.4	1
4	7	Q14081	29.5	1
1	1	Q5T7N2	1	1
2	3	Q9NZH7	8.5	1
1	1	A4D1T6	1.1	1
6	6	B4DWQ5	2.2	1
1	1	A0A024R8E5	0.5	1
2	2	A0A2R8Y5P9	0.4	1
1	1	A0A384N5W4	0.7	1
1	2	A0A3B3IS60	5.1	1
3	3	A0A590UJJ6	5.8	1
2	3	K7ELE3	7.1	1
1	1	A0A5C2G9Q1	8	1
1	1	A0A669KBA8	0.8	1
3	3	J3KPS0	5.1	1
1	1	Q4FZB7	2.9	1
1	1	A0A494BZV4	2	1
3	3	L0R8D2	14.4	1
1	1	P12524-2	3.3	1
1	1	A0A087WWM5	1.1	1
1	1	E9PKS9	8.7	1
2	3	A0A0S2Z4Z8	1.8	1
1	1	Q6ZP15	6.2	1
2	2	Q8N2Y8	1.6	1
2	2	A0A0C4DG40	0	1
3	4	B2RCA1	0.7	1

1	1	B3KRS6	3.4	1
1	1	H3BPH4	7.7	1
1	2	P01040	27.5	1
3	3	A6X8I4	2.9	1
2	2	A0A6Q8PF43	11.3	1
2	2	A0A6Q8PGG9	0.5	1
3	5	Q4G193	19.5	1
1	1	A0A8I5KSX0	0.8	1
2	2	H7BZD5	0.4	1
2	2	H0YLF3	16.9	1
3	4	A0A024QZZ7	7.1	1
2	2	A0A0F6MY87	0.8	1
1	1	A7LNJ1	1.4	1
1	1	A0A0X9USN3	15.8	1
1	1	A0A140VK80	2.4	1
5	6	Q9BZZ5	1.6	1
1	1	H0Y6Z0	8.2	1
2	2	Q96G01	1.8	1
1	1	A0A126LB36	1.2	1
1	1	B4DMD8	1.9	1
1	2	B3KRY9	4.2	1
1	2	B4DNN4	1.7	1
2	2	Q96RW7	0.3	1
1	1	J3QLK7	2.8	1
1	1	A8K6H6	1	1
2	2	B3KU69	3	1
6	8	O43147	1	1
1	1	Q8N3K9	0.2	1
1	1	Q9NQ89	1.2	1
1	2	A0A140VJI6	0.1	1
1	1	A6XNC9	4.4	1
5	5	Q86YW0	1.3	1
1	1	A0A024R643	0.8	1
1	1	A0A2H4QWS7	36.7	1
1	1	A0A7I2V490	1.6	1
2	2	G3V1X8	0.7	1
1	1	J3QQJ1	7.5	1
1	5	A0A024RAQ4	18	1
2	2	A0A1U9X7D6	2.6	1
1	1	Q9UBY0	1.2	1
1	1	A0A024R2V0	1.6	1
1	2	A0A024R889	0.6	1
1	1	Q06265-3	2.5	1
1	1	G8I2S8	5.6	1
3	4	Q6P5S2	2.7	1
1	1	A0A5F9ZHL6	1.2	1

1	1	A1L4G8	0.8	1
2	3	P0C091	1.3	2
1	1	A6NCT7	1.5	1
1	1	A0A8C8KBU6	1.3	1
1	1	P05109	11.8	1
1	2	A0A2R8Y4X0	3.4	1
1	1	Q59EX4	0.9	1
6	6	A0A140TA62	2.2	1
1	2	A0A494C128	2.5	1
1	1	A0A7P0T897	2.2	1
1	2	B4DDR2	2	1
1	1	A0A024R0R1	0.8	1
2	2	A0A590UJ10	0.5	1
2	2	Q8N1Q8	3.2	1
1	1	A0A0A0MSY6	1.8	1
1	1	A0A024R107	2.8	1
1	1	A0A024R6X7	3.6	1
1	1	A0A0A0MSH6	3.1	1
1	1	B2RBJ8	3.2	1
1	2	B4E395	5.7	1
1	1	E9PQ73	0.3	1
1	1	P17677	9.2	1
1	1	Q2VY69	2.3	1
1	1	Q8N8T0	7.9	1
1	1	B3KQD0	2.6	1
1	1	A0A087WXE9	3.5	1
1	1	B9EGB3	3.5	1
2	2	A0A0S2Z3H5	0.8	1
1	1	A8K4K0	1.8	1
3	3	B7Z999	1.5	1
1	2	H3BR51	5.4	1
1	1	A0A2U9QGI0	0.5	1
1	1	A0A494C0A7	1.5	1
5	6	A0A7L4X6S8	2.2	1
2	2	B3KQ41	2.1	1
1	1	A0A024RCR5	1.4	1
1	1	A0A0K0K1H8	1.5	1
1	1	F5GWR3	5	1
2	2	A0A140VK12	1.6	1
1	1	D6RJA6	2.9	1
1	1	Q5GH76	1.6	1
1	1	Q9NSI6	0.6	1
5	6	A0A0U1RQL2	3.6	1
1	1	A0A1U9X8D4	11.1	1
1	1	A4Z946	1.4	1
1	1	A0A096LNH7	2.5	1

1	1	B4DR79	3.3	1
1	1	B7Z217	0.8	1
1	1	H0YI54	19.5	1
1	1	P59665	9.5	1
1	1	A0A0S2Z508	1	1
1	1	A0A669KBJ0	1	1
1	1	A0A024R906	2.6	1
1	1	A0A2X0SF55	1.1	1
2	3	A0A087WWA5	1.7	1
2	3	B4DID2	8.7	1
1	1	H3BVF7	1.1	1
2	2	Q6AI22	0.3	1
1	1	A0A0A0MRA3	0	1
1	1	A4D2F6	1.4	1
1	1	B0I1S4	0.1	1
1	1	A0A024R017	6.9	1
1	1	A0A024RAL3	0.7	1
2	2	A0A087WYB4	9.3	1
1	1	A0A5C2FYH5	7.2	1
1	1	A0A024R8S5	1.9	1
1	1	B4DEV5	1.4	1
1	1	A0A024R5V1	0.9	1
1	1	A0A0A0MR51	3.1	1
1	1	B3KQF5	3.4	1
1	1	B4DTB2	2.5	1
3	5	B7ZML4	0.5	1
1	1	A0A146IHP0	1.8	1
1	1	A6NHR2	3	1
1	1	Q1KMD3	2	1
1	1	E5RHA8	6.9	1
1	1	P08922	0.4	1
1	1	Q6ZNV8	11.5	1
1	1	B5A927	7.4	1
1	1	Q14CC5	5.2	1
1	1	B2R713	1.5	1
1	1	B3KP66	5.9	1
1	1	B4DDW4	10.4	1
1	1	P20061	3.4	1
1	1	A0A024R5Z7	5.3	1
1	1	F6SA91	3.2	1
1	1	A1IGU5	2.2	1
1	1	A8K489	4.7	1
1	1	D4YW74	0.1	1
1	1	B4E1X6	1.3	1
1	1	A0A024R8D0	5.9	1
1	1	O43304	3.8	1

1	1	Q9H0C2	2.5	1
1	1	B4DYT8	2.4	1
1	1	A2IDD5	1.3	1
1	1	B3KND7	5.7	1
1	1	B7Z208	12.8	1
1	1	Q12913	1.3	1
1	1	A0A024R023	2.1	1
1	2	Q6ZP38	7	1
1	1	A0A3B3IUD7	0.3	1
1	1	B2R577	7.7	1
1	1	R4GMP5	37.3	1
1	1	E7EQM7	4.8	1
1	1	F8VW50	2.1	1
1	1	Q7Z442	0.3	1
1	1	A4D202	1.4	1
1	1	P29475-4	4.1	1
1	1	B4DQE2	6.7	1
1	1	B7Z6Y6	1.6	1
1	1	Q4EW64	87.5	1
1	1	A0A024R930	1.4	1
2	2	A0A024RC06	2.9	1
1	1	A0A0S2Z3G9	1.8	1
1	1	A0A5C2GBH9	10.3	1
1	1	G3V5R9	2	1
1	1	A0A0S2Z5F5	1.2	1
1	1	A0A1B0GVM6	7.9	1
1	1	A6PVK0	2	1
1	1	D6RFI4	3.4	1
1	1	A4D0Q4	11.3	1
1	1	C9JT30	2.8	1
1	1	A0A678ZHA3	7.4	1
1	1	A0A075B6T4	3.3	1
1	1	B4DN86	2.9	1
1	1	E3VSK7	7.9	1
1	2	B4DQZ7	1.4	2
1	1	Q49AP3	4	1
1	1	A0A1W2PPH1	1.6	1
1	1	A5YM46	1.2	1
1	1	D3DSM4	2	1
2	2	A0A024R732	1.6	1
1	2	A0A0A0MSI1	6.5	1
2	4	A0A126GVN0	2.2	1
1	1	V9HVX8	1.1	1
4	4	A5PL10	0.7	1
1	1	B7Z4Z6	9.1	1
1	1	Q5T953-2	9.2	1

1	1	A6NGG8	1.7	1
1	1	Q5T440	8.7	1
5	5	A0A024R3F4	0.6	1
1	1	A0JLQ4	6	1
1	1	B2R5Q5	2.1	1
1	2	Q7Z3G2	2.7	1
1	1	A5PKX5	2.2	1
1	1	A0A024RAY7	0.7	1
1	1	A0A024R5U8	4.2	1
1	1	A8MYS5	2.8	1
1	1	Q07283	0.8	1
1	1	Q8NCD3-2	2.7	1
1	1	B4DG49	6.8	1
2	3	A0A0S2Z652	1.2	1
1	1	B2RDG4	3.1	1
1	1	Q9UK58	4.9	1
1	1	B4DQ81	0.8	1
1	1	F2Z395	3.7	1
1	1	A8K2V5	2.1	1
1	1	Q8WXW3	1.1	1
1	1	O00420	3.5	1
1	1	B2R7D2	2.2	1
1	1	A0A140VKD0	0.5	1
1	1	A0A2X0SFR8	0.7	1
2	2	H0Y9G8	9.7	1
1	2	A0A024R188	1.4	1
1	1	A0A024QZT2	0.6	1
2	2	C9JKF1	1.8	1
1	1	D6RC71	9.4	1
1	1	Q6ZTR5	0.3	1
1	2	M0R2F0	22.6	1
1	1	A2VDJ1	3.1	1
1	1	D3U719	1.5	1
1	1	Q5T9C2	6.5	1
2	2	A0A8I5KXC3	1.1	1
1	1	A8K273	1.9	1
1	1	B3KQE9	5.5	1
1	1	A0A024RAF4	1	1
1	1	B4DFQ5	4	1
1	1	Q5T0W9	1.1	1
2	2	A0A2R8Y362	2	1
1	1	Q3LIF3	2.7	1
1	1	Q14571	0.4	1
1	1	A0A0B6XK12	8.9	1
1	1	Q2F3K6	14.7	1
1	1	C9JXP5	4.9	1

5	6	Q8NHP6	1.7	1
1	1	A0A087WYF6	0.8	1
1	1	A0A2R8Y4V0	0.7	1
1	1	A0A024R8C5	4.5	1
1	1	D6W641	4.5	1
4	4	B4DY32	2	1
1	1	A0A6Q8PFD1	14.4	1
1	2	A0A804HJD7	3	1
1	1	A0A2R8YDB8	1.2	1
1	1	Q99996-4	0.6	1
1	1	A0A087WW06	1.1	1
1	1	A0A087WUT8	1.8	1
1	1	D6RGG3	0.3	1
1	1	A0A2X0SYS0	0.4	1
1	1	A0A2P9DTZ0	0.8	1
1	1	A0A1U9X8U9	1.7	1
1	1	E9PQS0	3.1	1
2	2	Q9C000	0.4	1
1	1	A4FUT8	2.2	1
2	2	A0A024R4D3	1	1
1	1	F5GZI3	1.8	1
1	1	A0A024R7T5	3.4	1
1	1	A0A024RAY1	2.1	1
1	1	C9JXJ9	5.1	1
1	1	B4E3F0	12.5	1
1	1	B4E1R5	6.7	1
1	1	Q9NR99	0.7	1
1	1	A6NL88	1.4	1
1	1	A0A140VJN4	2.8	1
1	1	B3KS48	1.3	1
1	1	A0A1B0GTV9	0.7	1
1	1	A0A075B6G3	0.4	1
2	2	A0A0K0K1I0	3.1	1
1	1	Q15572	1.3	1
1	1	A0A1S5UYZ9	1	1
1	1	A0A087WV00	0.7	1
1	1	A0A0G2JLI4	2.9	1
1	1	A0A2X0SSM1	1.3	1
1	1	Q9NVW2	1.6	1
1	1	A0A0F6QNT9	1.8	1
1	1	A4UU13	1.2	1
1	1	O60747	4.4	1
1	3	A0A0A0MR67	0.3	1
1	1	A8MXY4	1.1	1
1	1	A0A087X170	1.1	1
1	1	Q05C31	3.5	1

1	1	A0A5C2GH48	23.3	1
1	1	A0A2X0SFB0	0.7	1
1	1	A0A3B3IST1	11.4	1
1	1	A0A0C4DG82	1.3	1
1	1	H0YL99	7.6	1
1	1	A4D1D2	1	1
2	2	A0A068F7M9	0.7	1
1	1	A0A087WV86	2.6	1
1	1	H0YMT6	3.6	1
1	1	A0A2R8YFH5	1	1
1	1	A0A3B3ISX0	8.8	1
1	1	B7Z4C3	3	1
1	1	Q58FG1	4	1
1	1	E9PJB2	9.7	1
1	1	A0A1B0GVP4	0.6	1
1	1	B4DTD8	1.2	1
1	1	A0A024R4L7	1.5	1
1	1	A0A0A0MRQ7	7.3	1
1	1	O75164-2	1.6	1
1	1	P42025	9	1
1	1	B3KPV9	1.9	1

SupplementaryTable 2. Characterization and identification of intensity values presented by all study participants of the three proteins with differences between groups

CKD-ESRD group (n=10)										Healthy group (n=10)										Columns Protein Observed	Spectra Protein Observed	Database Accession	%AA Coverage	Distinct Peptides	VIP Score
0.00e+000	0.00e+000	0.00e+000	0.00e+000	0.00e+000	0.00e+000	0.00e+000	0.00e+000	0.00e+000	0.00e+000	1.40e+005	0.00e+000	2.90e+005	3.48e+004	1.02e+005	0.00e+000	0.00e+000	0.00e+000	2.82e+005	3.41e+004	6	8	O43147	1	1	2.42174
0.00e+000	0.00e+000	0.00e+000	0.00e+000	0.00e+000	0.00e+000	0.00e+000	0.00e+000	0.00e+000	0.00e+000	0.00e+000	0.00e+000	9.60e+004	1.93e+005	0.00e+000	5.58e+004	0.00e+000	8.54e+004	0.00e+000	1.64e+005	5	6	Q9BZZ5	1.6	1	2.5344
0.00e+000	0.00e+000	0.00e+000	0.00e+000	0.00e+000	0.00e+000	0.00e+000	0.00e+000	0.00e+000	0.00e+000	0.00e+000	0.00e+000	9.60e+004	9.63e+004	0.00e+000	5.58e+004	0.00e+000	8.54e+004	0.00e+000	1.64e+005	5	5	Q86YW0	1.3	1	2.5473
